# Effect of Global ATGL Knockout on Murine Fasting Glucose Kinetics

**DOI:** 10.1155/2015/542029

**Published:** 2015-07-05

**Authors:** Margarida Coelho, Patricia Nunes, Vera M. Mendes, Bruno Manadas, Arend Heerschap, John G. Jones

**Affiliations:** ^1^CNC—Center for Neuroscience and Cell Biology, University of Coimbra, Coimbra, Portugal; ^2^MRC National Institute for Medical Research, London, UK; ^3^Department of Radiology, Radboud University Nijmegen Medical Centre, Nijmegen, Netherlands; ^4^Portuguese Diabetes Association (APDP), Lisbon, Portugal

## Abstract

Mice deficient in adipose triglyceride lipase (ATGL^−/−^) present elevated ectopic lipid levels but are paradoxically glucose-tolerant. Measurement of endogenous glucose production (EGP) and Cori cycle activity provide insights into the maintenance of glycemic control in these animals. These parameters were determined in 7 wild-type (ATGL^+/−^) and 6 ATGL^−/−^ mice by a primed-infusion of [U-^13^C_6_]glucose followed by LC-MS/MS targeted mass-isotopomer analysis of blood glucose. EGP was quantified by isotope dilution of [U-^13^C_6_]glucose while Cori cycling was estimated by analysis of glucose triose ^13^C-isotopomers. Fasting plasma free fatty-acids were significantly lower in ATGL^−/−^ versus control mice (0.43 ± 0.05 mM *versus* 0.73 ± 0.11 mM, *P* < 0.05). Six-hour fasting EGP rates were identical for both ATGL^−/−^ and control mice (79 ± 11 *versus* 71 ± 7 *μ*mol/kg/min, resp.). Peripheral glucose metabolism was dominated by Cori cycling (80 ± 2% and 82 ± 7% of glucose disposal for ATGL^−/−^ and control mice, resp.) indicating that peripheral glucose oxidation was not significantly upregulated in ATGL^−/−^ mice under these conditions. The glucose ^13^C-isotopomer distributions in both ATGL^−/−^ and control mice were consistent with extensive hepatic pyruvate recycling. This suggests that gluconeogenic outflow from the Krebs cycle was also well compensated in ATGL^−/−^ mice.

## 1. Introduction

The ectopic accumulation of lipids in tissues such as the skeletal muscle and liver is highly implicated in the pathogenesis of insulin resistance and glucose intolerance. In skeletal muscle, elevated levels of intramyocellular lipids are strongly associated with impaired rates of insulin-stimulated whole-body glucose disposal [1]. In the liver, excessive intracellular lipid levels are associated with impaired insulin-mediated suppression of glucose production [2]. However, there are settings where elevated tissue lipid levels do not confer insulin resistance, most notably the “athlete's paradox” where highly trained athletes have elevated levels of intramyocellular triglyceride yet are highly insulin sensitive [3, 4]. A similar paradox is presented in subjects with loss of function mutations in the adipose triglyceride lipase (ATGL) gene [5]. ATGL is the rate-limiting enzyme for triglyceride catabolism in most cells and tissues and a lack of ATGL activity results in a dyslipidemic phenotype with augmented visceral adipose tissue and extensive ectopic triglyceride accumulation in pancreas and skeletal muscle [5]. These subjects, though glucose intolerant on account of a limited insulin secretory response, nevertheless show normal whole-body insulin sensitivity [5]. This profile is recapitulated in the ATGL knockout (ATGL^−/−^) mouse model. These mice show augmented adipose tissue mass, as well as extensive ectopic triglyceride accumulation in heart, liver, and skeletal muscle [6, 7]. The excessive myocardial triglyceride accumulation is associated with the development of severe cardiac insufficiency at about 12 weeks of life [6]. Despite this excessive adiposity, ATGL^−/−^ mice are glucose tolerant and insulin sensitive and the basis of this paradox remains poorly understood.

**Figure 1:**
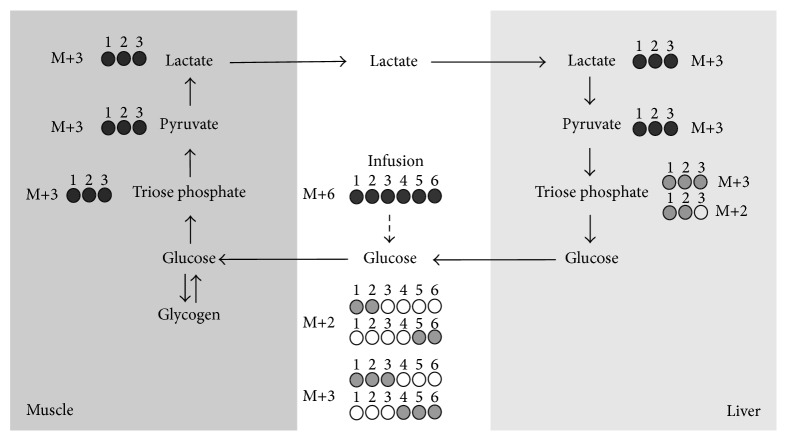
Schematic of plasma glucose ^13^C-isotopomer formation following infusion of [U-^13^C_6_]glucose (represented by black closed circles), its initial conversion to [1,2,3-^13^C_3_]pyruvate and lactate via glycolysis, and gluconeogenic conversion of this isotopomer to form M+2 and M+3 glucose isotopomers (shown in grey).

In the basal fasting state, glucose homeostasis is dependent on a tight control of endogenous glucose production (EGP) and efficient peripheral glucose disposal. The curtailment of triglyceride hydrolysis in ATGL^−/−^ mice can potentially modify both liver and muscle glucose metabolism, ultimately contributing to improved glucose tolerance. In skeletal muscle, a reduction in free-fatty acid (FFA) levels due to attenuation of triglyceride hydrolysis can potentially favor glucose oxidation in accord with the postulations of the Randle cycle [8], thereby promoting peripheral glucose disposal. Meanwhile, a restriction in availability of systemic FFA and glycerol results in restricted gluconeogenesis from glycerol as well as pyruvate, the latter being highly dependent on *β*-oxidation for ATP and reducing equivalents. It is not known to what extent glucose tolerance in the ATGL^−/−^ mouse is explained by improved peripheral glucose oxidation versus restriction of gluconeogenesis. Therefore, the aim of this study was to compare EGP and peripheral glucose disposal in ATGL^−/−^ versus wild-type (WT) mice. This was achieved by ^13^C-isotopomer analysis of plasma glucose following a primed-infusion of [U-^13^C_6_]glucose: a tracer that provides a measurement of EGP by isotope dilution, as well as a measure of glucose carbon recycling via the Cori cycle, a measure of the sparing of glucose oxidation by peripheral tissues as illustrated by Figure 1. Plasma glucose ^13^C-isotopomers from blood spots were quantified by a novel and sensitive isotopomer-targeted LC-MS/MS analysis. Unlike GC-MS methods that require sample derivatization [9], this analysis was achieved following a simple and rapid sample extraction procedure.

## 2. Materials and Methods

All experimental procedures were approved beforehand by the local Animal Ethics Committee of the Radboud University Nijmegen Medical Center (RUNMC) (Nijmegen, Netherlands). The mice used in this project were a kind gift of Professor Rudolf Zechner from Graz University (Graz, Austria). ATGL^−/−^ mice were bred from heterozygous ATGL (ATGL^+/−^) in a mixed genetic background C57BL/6J and 129Ola as described previously [6]. Mice were fed* ad libitum* a standard chow diet and were housed in 12 h/12 h light-dark cycle with controlled temperature (22–24°C). Six whole-body knockout ATGL^−/−^ mice and seven ATGL^+/−^ littermates (referred to hereafter as wild-type (WT) mice) were used for this study. Animals were fasted for 6 h and anesthetized with 1.5% isoflurane given by a gas mixture (2 : 1 O_2_/N_2_O) through a facial mask. They were administered intravenously a primed-constant infusion of [U-^13^C_6_]glucose consisting of a 6.25 *μ*mol/kg priming dose followed by 5 *μ*mol/kg/min constant infusion for 120 min.

Blood spots were collected directly onto Whatman number 6 filter paper at 105, 110, and 115 min. For a subgroup of animals, blood spots were collected before the start of tracer infusion in order to obtain background ^13^C levels. Six mm blood spot disks were punched; then 50 *μ*L of internal standard (IS) solution consisting of 30 *μ*M [U-^13^C_6_,U-^2^H_7_]glucose in distilled water and 450 *μ*L of EtOH were added. Samples were sonicated in a bath for 45 min at room temperature and then centrifuged for 5 min at 13,900 ×g. The supernatant was evaporated at 60°C and resuspended in 50 *μ*L of H_2_O. Samples were sonicated with a cup-horn at 40%, for 2 min, with pulses of 1 sec and pauses of 1 sec. Samples were subsequently purified by solid phase extraction (SPE) using reversed phase packed zip-tips and ACN/H_2_O as the mobile phase.

### 2.1. LC-MS/MS Analysis

The LC system used for all analyses was an Ultimate 3000 LC system (Thermo Scientific, Dionex). The MS used was a hybrid triple quadrupole/linear ion trap 4000 QTRAP LC-MS/MS system equipped with an electrospray ionization (ESI) Turbo V ion spray source (ABSciex). The software operating the LC system was the Chromeleon 6.80 (Thermo Scientific, Dionex) and the MS system was controlled by the Analyst 1.5.1 (ABSciex).

Chromatographic separation was performed on a Luna NH_2_ 3 *μ*m, 100 Å, 150 × 2.00 mm column. The flow rate was 150 *μ*L/min and running time for each sample was 15 min. Samples were eluted with an ACN/H_2_O gradient starting at 80%/20%, reaching 60%/40% at 5 min and 20%/80% by 15 min. The retention time for glucose was approximately 8 min. After sample injection, one blank (H_2_O) of 4 min running and afterwards a second blank (H_2_O) of 8 min running were injected. The sample injection was 1 *μ*L, while for the blanks 19 *μ*L was injected.

Samples were analyzed at 150 *μ*L/min by ESI. The ionization source operated in the negative mode at an ion spray voltage of 4500 V, with nebulizer gas 1 pressure set to 35 psi, nebulizer gas 2 set to 30 psi, and a temperature of 450°C. The 4 min blank was operated in the positive mode, while the 8 min blank program was operated in the negative mode. Glucose enrichments were quantified using the multiple reaction monitoring (MRM) triple quadrupole scan mode. The following MS parameters were maintained for all transitions: collision gas 6 psi, curtain gas 30 psi, collision cell exit potential −8 eV, entrance potential −4 eV, dwell time 100 ms, and declustering potential −50 eV. The collision energy was optimized for each transition. MRM transitions for monitoring [U-^12^C_6_]glucose were 179/89, for [1,2-^13^C_2_]glucose 181/89 and 181/91, for [1,2,3-^13^C_3_]glucose 182/89 and 182/92, and for [U-^13^C_6_]glucose 185/92. Finally, for the [U-^13^C_6_, U-^2^H_7_]glucose internal standard, the selected transition was 192/94. Peak areas were integrated using the Multiquant 2.1.1 software (ABSciex).

Excess enrichments of glucose ^13^C-isotopomers were quantified from calibration curves prepared with known ratios of unenriched glucose to [1,2-^13^C_2_]glucose, [1,2,3-^13^C_3_]glucose, and [U-^13^C_6_]glucose. The amount of glucose ^13^C-isotopomers measured from the calibration solutions spanned the range of measured concentrations from the blood samples. Analysis of single and combined ^13^C-isotopomer standards indicated no significant cross-contamination of M+6, M+3, and M+2 ^13^C-mass isotopomer between the different ^13^C-enriched glucose standards.

### 2.2. Quantifying Endogenous Glucose Production and Cori Cycle Fluxes

Enrichment of plasma glucose from infused [U-^13^C_6_]glucose (*E*_*p*_) was determined from the M+6 abundance of plasma glucose. The rate of appearance (*R*_*a*_) of plasma glucose and EGP were calculated as follows:

(1) Glucose  Ra=EiEp×i,EGP=Ra−i,

where *i* is infusion rate of [U-^13^C_6_]glucose in *μ*mol/kg/min and *E*_*i*_ is the percent enrichment of infusate [U-^13^C_6_]glucose. The units of *R*_*a*_ and EGP were reported as *μ*mol glucose/kg body weight/min. The rate of peripheral glucose disposal (*R*_*d*_) was assumed to be equal to *R*_*a*_. For quantifying Cori cycle activity, the following equations were used:

(2) Fraction  of  glucose  Ra  undergoing  Cori  Cycling=1.5×0.5×M+2+M+3M+6,Absolute  Cori  cycling  flux=Fractional  Cori  cycle  flux×Rd,

where the fraction of glucose *R*_*d*_ undergoing Cori cycling is given as a percentage and the absolute Cori cycle flux is reported as *μ*mol/kg/min.

Plasma glucose M+6 enrichment was considered as the precursor pool while the sum of plasma glucose M+2 and M+3 isotopomer enrichments was assumed to be the products following passage of the M+6 species through the Cori cycle. The M+3 species detected by the 182/92 LC-MS/MS transition was assumed to represent the sum of [1,2,3-^13^C_3_]glucose and [4,5,6-^13^C_3_]glucose isotopomers while the M+2 species detected by the 181/91 transition was assumed to represent the sum of [1,2-^13^C_2_]glucose and [5,6-^13^C_2_]glucose isotopomers (Figure 1). These four species represent ~85–90% of glucose isotopomers that are formed during recycling of [U-^13^C_6_]glucose [10, 11]. Gluconeogenesis also results in an approximately ~1.5-fold dilution of ^13^C-enrichment via Krebs cycle carbon exchange; hence a correction factor of 1.5 was incorporated into the equation [10, 11]. Finally, since one M+6 glucose generates two recycled molecules containing one or the other of the M+3 and M+2 daughter isotopomers, for calculating the recycling fluxes, the sum of M+3 and M+2 isotopomer enrichments was divided by 2 to maintain the correct stoichiometry between the plasma glucose precursor and recycled glucose product.

## 3. Results

**Figure 2:**
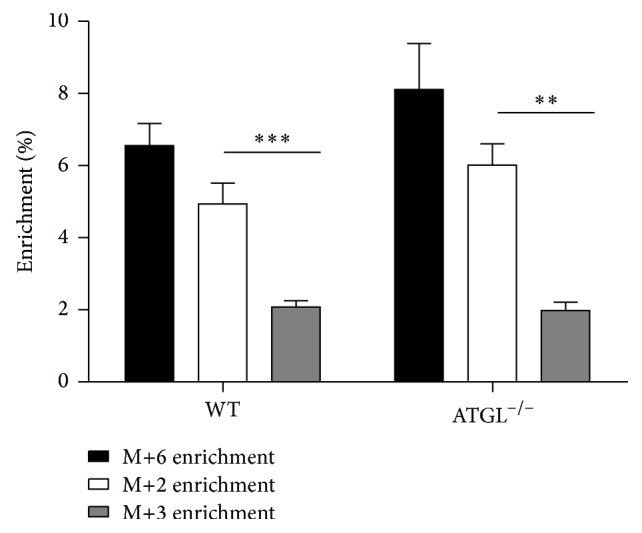
Percent enrichment of [U-^13^C_6_]glucose (M+6), [1,2-^13^C_2_] + [5,6-^13^C_2_]glucose (M+2), and [1,2,3-^13^C_3_] + [4,5,6-^13^C_3_]glucose (M+3) in WT (*n* = 7) and ATGL^−/−^ mice (*n* = 6). Data are expressed as mean ± SEM. ^*∗*^ *P* < 0.05.

The physiological characteristics of ATGL^−/−^ and WT mice are shown in Table 1. Both groups of mice were statistically not different in age, but the ATGL^−/−^ group tended to be younger. This was due to some spontaneous deaths due to heart failure occurring in this group at about 8 weeks of age in addition to some mortalities during the tracer administration protocol, also likely related to heart failure. Food intake was similar for both groups. After 6 h of fasting, both groups had equivalent fasting plasma glucose and plasma triglyceride levels but ATGL^−/−^ mice had significantly lower levels of plasma FFA at 6 h of fasting compared to WT mice. The glucose M+6, M+3, and M+2 isotopomer distributions are summarized in Figure 2. For a subset of animals (4 ATGL^−/−^ and 2 WT), a blood sample was collected immediately before [U-^13^C_6_]glucose infusion to determine background levels of M+2, M+3, and M+6. Based on our detection limit criteria (signal-to-noise ratio ≥3), no signals from any of these isotopomers were observed in any of the samples. For blood that was collected after [U-^13^C_6_]glucose infusion, most of the variance in ^13^C-isotopomer enrichments reported for each group arose from differences between animals, with ATGL^−/−^ mice showing somewhat higher variance than WT mice. Thus, the variability in glucose mass isotopomer enrichments between the 105-, 110-, and 115-minute blood samples for each animal was low, with the mean coefficient of variation being 7% for the parent M+6 isotopomer and 8% and 11% for the daughter M+2 and M+3 isotopomers, respectively. For both ATGL^−/−^ and WT mice, the distribution of the recycled glucose ^13^C-isotopomers was heavily skewed toward M+2 over M+3 species (Figure 2). An excess of M+2 over M+3 isotopomer distribution within the triose moiety of glucose was also previously reported in rats infused with [U-^13^C]lactate [12]. Also, an excess of [1,2-^13^C_2_]glucose over [1,2,3-^13^C_3_]glucose isotopomers has been quantified by ^13^C NMR in mice administered with [U-^13^C]propionate [13, 14], an alternative gluconeogenic substrate that, like [U-^13^C]lactate, is also metabolized to glucose via the hepatic Krebs cycle and anaplerosis.

Flux estimates derived from the ^13^C glucose isotopomer distributions are shown in Table 2. No differences in either glucose *R*_*a*_ or EGP rates were found between WT and ATGL^−/−^ mice. Our estimated EGP values are higher compared to values of ~50 *μ*mol/kg/min that were previously reported for fasted WT mice that were chronically catheterized and fasted for 26 h [15]. It is known that EGP declines during the progression of fasting [16]; hence a 6 h fasted mouse would be expected to have higher EGP rates compared to a 26 h fasted mouse. WT and ATGL^−/−^ mice also had similar rates of fractional and absolute Cori cycle fluxes. Cori cycling accounted for the majority (~80%) of glucose utilization indicating that peripheral glucose oxidation was highly spared in both groups of mice.

## 4. Discussion

There is a strong association between elevated ectopic lipid levels and insulin resistance in both humans and rodents [17–19]. Metabolites related to triglyceride-fatty acid interconversion such as fatty-acyl CoA, diacylglycerol, and ceramides have been shown to disrupt insulin signaling [20–23] and have therefore been hypothesized as the causative agents of insulin resistance in the setting of elevated ectopic lipid. However, this view is challenged by the ATGL^−/−^ mouse since it has extensive levels of ectopic lipids in insulin-sensitive tissues such as skeletal muscle, heart, and liver but is nevertheless glucose tolerant. In contrast, when ectopic lipid depots are induced in WT mice via high-fat and/or high-sugar feeding, they develop insulin resistance and glucose intolerance [24, 25]. In the basal fasted state, insulin sensitivity is primarily defined by the matching of hepatic glucose production to peripheral glucose uptake. With diet-induced dyslipidemia and ectopic lipid accumulation, there is a well characterized upregulation of fasting EGP [26, 27], primarily due to the impaired control of hepatic gluconeogenesis. This so-called hepatic insulin resistance is a prominent and early feature of diet-induced glucose intolerance and is strongly associated with elevated hepatic lipid levels. Meanwhile, the accumulation of lipid in skeletal muscle, albeit slower than the development of hepatic steatosis, is nevertheless accompanied by a reduction in insulin-stimulated glucose disposal [28].

The features of fasting glucose production and peripheral disposal that explain the high glucose tolerance in ATGL^−/−^ mouse remain unclear. In the initial study on the effect of ATGL knockout on metabolic status and insulin sensitivity by Haemmerle et al. [6], ATGL^−/−^ and WT controls had similar levels of fasting plasma glucose and insulin. This suggests that ATGL^−/−^ and WT mice had similar insulin sensitivity under fasting conditions, at least as assessed by fasting insulin and glucose levels. However under fed conditions and also following a glucose load, ATGL^−/−^ mice revealed higher insulin sensitivity compared to WT mice [6]. Since insulin-stimulated disposal of glucose into peripheral tissues assumes a much greater role in glycemic control under fed compared to fasted states, this suggests that the higher insulin sensitivity of ATGL^−/−^ mice is more related with peripheral rather than hepatic insulin actions. In support of this, Kienesberger et al. reported that* in vivo* ATGL^−/−^ skeletal muscle insulin signaling was improved with increased insulin receptor substrate 1 and Akt phosphorylation, PI3K and Akt activities, and GLUT4 protein expression [29]. Interestingly, hepatic insulin signaling was unchanged or impaired in ATGL^−/−^ mice [29], suggesting a more prominent role of peripheral over hepatic insulin actions in explaining their high insulin sensitivity. The role of extrahepatic tissues in determining whole-body insulin sensitivity of ATGL^−/−^ mice becomes even more prominent under increased energetic demand since they are more dependent on glucose as an oxidative fuel. Thus at rest, ATGL^−/−^ skeletal muscle ATP and other high-energy phosphate levels were comparable to the littermates and even upon high intense electrostimulation, the muscle oxidative capacity was not compromised [7]. However, ATGL^−/−^ mice showed significantly lower muscle glycogen levels both at rest and after electrostimulation, further supporting an increased demand for carbohydrate oxidation [7]. Similarly, Schoiswohl et al. showed that exercised ATGL^−/−^ mice presented hepatic glycogen reserves which were severely depleted [30]. This is consistent with an increased mobilization of glucose for skeletal muscle oxidation in response to insufficient fatty acid availability [30].

Conversely, the muscle-specific ATGL knockout, which recapitulates the high intramyocellular triglyceride level of the global ATGL^−/−^, did not have differences in oxidative substrate selection, glucose homeostasis, or peripheral insulin sensitivity compared to control mice [31] while resting muscle oxidative phosphorylation and oxidative capacity was not compromised in global ATGL^−/−^ mice [7]. Nevertheless, when exercised, ATGL^−/−^ mice showed limited generation of FFA while at the same time hepatic glycogen reserves were severely depleted [30]. This is consistent with an increased mobilization of glucose for skeletal muscle oxidation in response to insufficient fatty acid availability [30]. Furthermore, ATGL^−/−^ mice showed significantly lower muscle glycogen levels both at rest and after electrostimulation, further supporting an increased demand on carbohydrate reserves for muscle energy utilization [7]. Mice that underwent liver-selective ATGL knockdown developed steatosis following both normal and high-fat feeding but were protected against glucose intolerance and hyperinsulinemia during high-fat feeding [32]. While hepatic insulin signaling in response was not modified by hepatic ATGL knockdown, expression of gluconeogenic enzymes was decreased in both normal and high-fat feeding settings [32] suggesting reduced capacity for gluconeogenesis. Moreover, hepatic fatty acid oxidation was found to be impaired in ATGL^−/−^ mice [33], which, by restricting the availability of ATP and reducing equivalents, would also constrain gluconeogenesis from pyruvate precursors.

How do our measurements of fasting glucose kinetics reconcile with these previous studies? At 6 h of fasting, we found a significantly reduced level of plasma FFA in ATGL^−/−^ mice compared to WT, suggesting impairment of fasting whole-body lipolysis. The concentration of plasma FFA has been shown to exert strong and acute control of gluconeogenic flux [34–36], but the reduced availability of FFA in ATGL^−/−^ mice did not appear to compromise EGP fluxes, at least at 6 hours of fasting. We found that peripheral glucose metabolism was largely directed towards the Cori cycle, with the majority of glucose carbons being recycled. This suggests that skeletal muscle glucose oxidation, at least at rest, was not significantly enhanced in ATGL^−/−^ mice but was instead highly spared to the same extent as in wild-types. Presumably, the production of FFA by other lipases, for example,* spillover,* or the catabolism of other substrates as amino acids was sufficient to maintain the energy demands of skeletal muscle, at least in the resting state. A possible confounding factor in our study is the fact that the body weights of the ATGL^−/−^ mice were not significantly higher than the WT (due primarily to increased adiposity), as has been consistently reported in previous studies, for example, [6, 7]. This may be related to the tendency for the ATGL^−/−^ mice to be younger than their WT littermates as previously described. Intramyocellular lipid (IMCL) levels were assayed* in vivo* using ^1^H magnetic resonance spectroscopy in the tibialis anterior muscle of 3 ATGL^−/−^ and 4 ATGL^+/−^ mice of this study. ATGL^−/−^ mice had levels of IMCL relative to total creatine of 3.6 ± 0.44 versus 0.71 ± 0.80 in ATGL^+/−^ mice. Although the number of mice used for this analysis was limited, the data supports the characteristic ectopic lipid accumulation in ATGL^−/−^ mice.

Whether Cori cycle and EGP fluxes are similarly sustained in ATGL^−/−^ mice during longer fasting periods or during high-energy demanding states, for example, exercise, remains to be investigated. Presumably, the production of FFA by lipases other than ATGL was sufficient to maintain the energy demands of skeletal muscle, at least in the resting state.

**Figure 3:**
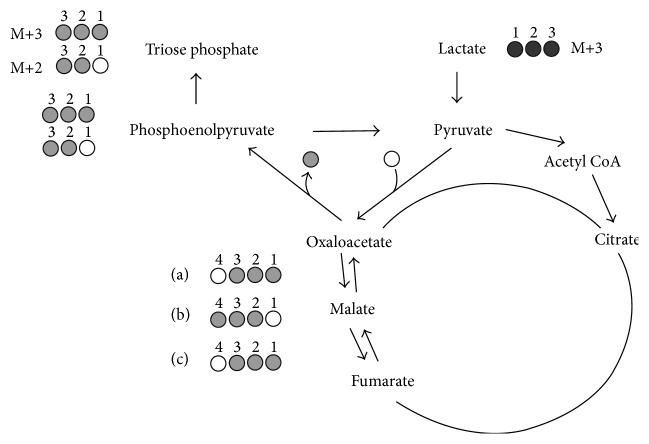
Generation of [2,3-^13^C_2_]triose-P (M+2) and [1,2,3-^13^C_3_]triose-P (M+3) isotopomers from [1,2,3-^13^C_3_]lactate via gluconeogenesis. The intermediate isotopomers of oxaloacetate are also shown. In the absence of pyruvate cycling and with extensive exchange of oxaloacetate with malate and fumarate, equal amounts of oxaloacetate isotopomers (a) and (b) are formed that result in the generation of equal amounts of M+2 and M+3 triose-P isotopomers. With pyruvate cycling, equal proportions of isotopomers (a) and (b) are converted to isotopomer (c). Since isotopomer (b) is the sole precursor of M+3 triose-P while isotopomers (a) and (c) both contribute to M+2 triose-P, this results in an excess of M+2 over M+3 triose-P.

In addition to providing estimates of EGP from isotope dilution of [U-^13^C_6_]glucose, LC-MS/MS also provided precise quantification of M+3 and M+2 triose isotopomers associated with Cori cycling. Their abundances were quantified from specific calibration curves prepared with [1,2-^13^C_2_]glucose and [1,2,3-^13^C_3_]glucose. The fragmentation mechanism that yields the 181/91 and 182/92 MS/MS transitions cleaves the glucose molecule into two fragments of identical mass representing carbons [1,2, 3] and [4,5, 6]. Thus, the abundance of M+2 and M+3 isotopomers from both triose halves of the glucose molecule was reported. For both WT and ATGL^−/−^ mice, we observed a significantly higher abundance of the glucose M+2 over the M+3 isotopomer. As illustrated by Figure 3, this can be explained by an excess production of [2,3-^13^C_2_]triose-P over [1,2,3-^13^C_3_]triose-P resulting from pyruvate cycling and is concordant with high activities of pyruvate cycling that have been described in fasted mice [13, 14], rats [37], and humans [38–40]. ^13^C-isotopomer studies of glucose enriched from [U-^13^C]propionate have shown that the formation of other possible M+2 triose isotopomers (i.e., [1,2-^13^C_2_]triose-P and [1,3-^13^C_2_]triose-P) is low relative to that of [2,3-^13^C_2_]triose-P, [13, 14]. Therefore, the observed excess of M+2 over M+3 primarily involves the “first pass” isotopomers described in Figures 1 and 3.

Previous estimates of M+2 and M+3 enrichments from [U-^13^C_6_]glucose recycling in rats and humans have revealed variable levels of M+2 and M+3 isotopomer products following [U-^13^C_6_]glucose infusion. In 24 h fasted rats infused intraduodenally with substrate levels of [U-^13^C_6_]glucose, a ~40% excess of M+2 to M+3 was found for both glucose and glycogen [41]. However, in another study where rats were infused intragastrically with [U-^13^C_6_]glucose, a small excess of M+3 over M+2 glucose was reported in both plasma glucose and hepatic glycogen. A study of healthy humans fasted for periods ranging from 12 to 40 h showed an excess of M+3 to M+2 in 12 and 20 h fasted individuals but a 2-fold excess of M+2 over M+3 in 40 h fasted subjects [42]. In a study of three 65 h fasted subjects, there was a modest excess of M+2 over M+3 for two of the subjects, while the third subject had a modest excess of M+3 over M+2 [43]. However, these human measurements were acknowledged to have limited precision and specificity due to low ^13^C-enrichment levels [42] while specific glucose M+2 and M+3 isotopomers were not independently calibrated in any of these aforementioned human or animal studies. As with previous GC-MS and NMR-based analyses of [U-^13^C]glucose enrichment and Cori cycling, our approach is ultimately limited by the precision of the isotopomer quantifications, in particular the recycled M+2 and M+3 species whose levels are typically lower than the parent [U-^13^C]glucose while at the same time background contributions, in particular to the M+2 species, are higher. Also, this approach does not provide information on other gluconeogenic precursors such as fructose and glycerol, which, in addition to glycogenolysis, could influence the fractional contribution of Cori cycling to EGP.

In summary, we quantified glucose appearance and recycling fluxes in 6 h fasted ATGL^−/−^ mice. EGP rates were similar to WT controls indicating that hepatic gluconeogenesis was well controlled. As for WT controls, the majority of glucose that underwent peripheral metabolism was spared from oxidation. This indicates that, under these resting conditions, there was sufficient availability of other substrates, presumably including fatty acids, for muscle energy generation. Finally, resolution of recycled glucose isotopomers by MS/MS revealed a distribution consistent with active hepatic pyruvate cycling for both ATGL^−/−^ and WT mice.

## Figures and Tables

**Table 1:** Physiological characteristics for WT and ATGL^−/−^ mice. Data are expressed as mean ± SEM. ^*∗*^ *P* < 0.05, ^*∗∗*^ *P* < 0.01.

Physiological parameters	WT	ATGL^−/−^
Age (weeks)	9.9 ± 0.4	7.8 ± 2.2
Body weight (g)	26.3 ± 1.1	23.4 ± 1.2
Liver index (liver wt/body wt)	0.0335 ± 0.0019	0.0267 ± 0.0019^*∗*^
Food intake (g/g of body wt/24 h)	0.123 ± 0.019	0.128 ± 0.010
Fasting glycemia	7.8 ± 0.5	7.8 ± 0.4
Plasma triglyceride (mg/dL)	103 ± 9	90 ± 4
Plasma free-fatty acids (mM)	0.734 ± 0.11	0.425 ± 0.051^*∗∗*^

**Table 2:** Glucose appearance and Cori cycling fluxes in WT and ATGL^−/−^ mice based on plasma glucose ^13^C-isotopomer analysis. Data are expressed as mean ± SEM.

Glucose fluxes	WT	ATGL^−/−^
Glucose *R*_*a*_ (*μ*mol/kg/min)	70.8 ± 6.7	78.7 ± 10.6
Glucose *R*_*d*_ (*μ*mol/kg/min)	70.8 ± 6.7	78.7 ± 10.6
EGP (*μ*mol/kg/min)	66.4 ± 6.6	73.1 ± 10.3
Absolute Cori cycle flux (*μ*mol/kg/min)	56.6 ± 5.5	66.9 ± 12.5
Fractional Cori cycle flux (% of *R*_*d*_)	79.9 ± 2.8	82.0 ± 6.6
